# Clinical effects of hemoperfusion combined with pulse high-volume hemofiltration on septic shock

**DOI:** 10.1097/MD.0000000000019058

**Published:** 2020-02-28

**Authors:** Laping Chu, Guangyao Li, Yafen Yu, Xiaoyan Bao, Hongyi Wei, Minhong Hu

**Affiliations:** a Department of Nephrology, Affiliated Hospital of Jiangnan University, Wuxi; b Department of Urology, The First Affiliated Hospital of Nanjing Medical University, Nanjing; c Department of Intensive Care Unit, Affiliated Hospital of Jiangnan University, Wuxi, Jiangsu Province, China.

**Keywords:** continuous veno—venous hemofiltration, hemoperfusion, pulse high-volume hemofiltration, septic shock

## Abstract

Sepsis can cause septic shock, multiple organ dysfunction and even death. The combination of different blood purification would be the certain trend in the treatment of sepsis.

This study was to evaluate the clinical effects of hemoperfusion (HP) combined with pulse high volume hemofiltration (PHVHF) on septic shock.

Thirty cases were involved in this study and were randomly divided into two groups: HP and PHVHF group (n = 15) and CVVH (continuous veno-venous hemofiltration) group (n = 15). Acute physiology and chronic health evaluation (APACHE) II scores, sequential organ failure assessment (SOFA) scores as well as biochemical changes were measured before and after the treatment. The levels of IL-6, IL-10, and TNF-α in plasma were assessed by ELISA before and after treatment for 2 and 24 h. The norepinephrine doses were also analyzed. The 28-day mortalities in both groups were also compared.

In both groups, body temperature (BT), respiratory rate (RR), white blood cells (WBC), C-reactive protein (CRP), Procalcitonin (PCT), lactic acid, serum creatinine, APACHE II scores and SOFA scores decreased after hemofiltration (*P* < .05). The HP&PHVHF group was superior to the CVVH group in CRP, APACHE II score (*P *< .01), and heart rate (HR), WBC, PCT, SOFA (*P* < .05). The doses of norepinephrine were also decreased after treatment (*P* < .01), with more reduction in the HP&PHVHF group (*P* < .05). After 24 h of treatment, the levels of IL-6, IL-10, and TNF-α decreased in both groups (*P *< .05), and the decrease was more significant in HP&PHVHF group (*P* < .05). In combined group, after 2 h of hemoperfison, there was a significant reduction in these inflammatory factors (*P* < .01). Combined therapy group's mortality was 26.7%, while CVVH group's was 40%.

HP combined with PHVHF has a significant effect on septic shock and can be an important therapy for septic shock.

## 1 Introduction

Sepsis, which has pathologic, physiologic abnormalities, is a clinical syndrome induced by infection. It can release various inflammation mediators and then cause septic shock, multiple organ dysfunction and even death, so it is a major public health concern. Analysis of an international database conveyed that 437 per 100,000 people per year suffered from sepsis between 1995 and 2015.^[1]^ Even with modern intensive care, severe sepsis remains a serious problem to overcome. The mortality of severe sepsis in hospital is about 44% and increases to 59% for septic shock.^[2]^ Recent research has focused on blood purification for the treatment of severe sepsis and septic shock. Given the different principles of each method of blood purification, it was reported that the combination of different methods would be the certain trend in the treatment of sepsis. Continuous veno-venous hemofiltration (CVVH) is effectively used for the treatment of severe sepsis, because it can remove inflammatory molecules continuously and slowly. At the same time it can stable hemodynamics.^[3]^ High-volume hemofiltration (HVHF), by increasing the amount of replacement fluid, can remove inflammatory factors more efficiently.^[4]^ Pulse high volume hemofiltration (PHVHF) is a modified model of HVHF. It carries out HVHF for a short period to improve plasma water exchange, and followed by CVVH for a long time to maintain the curative effect.^[5]^ The combination of hemoperfusion (HP) and PHVHF contains multiple principles of blood purification, including the strengthened absorption, and may have special advantages on the therapy of septic shock, but the clinical studies are scarce. This study aims to evaluate the effect of PHVHF combined with HP on the therapy of septic shock.

## 2 Patients and methods

### 2.1 Patients and groups

From January 2014 to December 2016, 30 patients from our intensive care unit suffering from septic shock were involved in this study with ethics committee approval of hospital. Informed consent was obtained from patient's immediate family. The diagnostic criteria of sepsis and septic shock was referred to the American College of Chest Physicians/Society of Critical Care Medicine Consensus Conference criteria (1992).^[6]^ The diagnosis of the 30 septic shock patients also meet the new diagnostic criteria of sepsis-3.^[7]^ Exclusion criteria were patients aged over 80, active bleeding, late stage of malignant tumor, death within 72 h after treatment and those who refuse blood filtration. The 30 patients were divided into two groups randomly: HP&PHVHF group (n = 15) and CVVH group (n = 15). In the HP&PHVHF group, septic shock was caused by respiratory infection in seven patients, urinary tract infection in two patients, cholangitis in four patients and abdominal infection in two patients. In the other group, septic shock was caused by respiratory in eight patients, urinary tract infection in one patient, cholangitis in four patients and abdominal infection in two patients.

### 2.2 Conventional treatment for septic shock

All the patients were admitted to intensive care unit (ICU) and were treated according to the international sepsis guidelines,^[8]^ such as early quantitative resuscitation, nutritional support, blood cultures before antibiotic therapy, broad-spectrum antimicrobials therapy, norepinephrine as the first-choice vasopressor to maintain pressure, support of dysfunctional organs and preventing the occurrence of stress-induced ulcer. Patients with acute respiratory failure underwent mechanical ventilation.

### 2.3 Hemofiltration technique

Using the Seldinger technique, an acute dual lumen catheter was inserted percutaneously either through the femoral vein or the right internal jugular for vascular access. Hemofiltration was carried out using a B. Braun Diapact CRRT machine and used a polyacrylonitrile hemofilter (B. Braun Diacap Acute L, Melsungen Germany). Blood flow rate was 200 to 250 mL/min. Replacement solution was added in pre-dilutionmode. Anticoagulation was obtained with low molecular weight heparin, and activated partial thromboplastin time was adjusted to 60 to 70 s. In combined group, concurrent PHVHF and HP were performed for the first 2 h. The HP cartridge preceded the hemofilter in the circuit. Hemoperfusion was undertaken with a resin cartridge (HA-330, Zhuhai Lizhu Group of Biological Material Co, Ltd. China) once a day. PHVHF was carried out with a daily schedule of HVHF(85 mL/kg/h)for 6 h followed by CVVH (35 mL/kg/h) for 18 h.^5^ In another group, CVVH (35 mL/kg/h) was performed for 24 h.

### 2.4 Data collection and measurements

Body temperature, respiratory rate, blood pressure and heart rate were recorded every half hour. Serum samples were collected at various time to measure WBC, CRP, Procalcitonin (PCT), creatinine, transaminase, bilirubin, lactic acid, electrolyte, and arterial blood gas before the treatment and after 24, 48, and 72 h. APACHE II scores and SOFA scores were used to assess the severity of the disease. The doses of norepinephrine required to maintain mean arterial pressure (MAP) above 85 mm Hg were also recorded every half hour. At the beginning of hemofiltration, after treatment for 2 and 24 h, blood samples were collected to measure the concentration of inflammatory factors such as IL-6, IL-10, and TNF-α by ELISA kits (Wuhan ColorfulGene biological technology Co., Ltd, China). Mortality was observed during the day on which patients received hemofiltration and at 28 days.

### 2.5 Statistical analysis

Results were expressed as means (SD)and analyzed with SPSS 19.0 statistical software. Enumeration data were analyzed using the student's *t* test. Mortality rate was analyzed through chi-square test. There is statistical significance when *P* < .05

## 3 Results

### 3.1 Outcome

In the present study, before the treatment, there were no significant differences in age, gender, clinical characteristics and severity of disease between the two groups (*P* > .05) (Table 1). Four patients in the HP&PHVHF group died in 28 days. The mortality was 26.7%. In CVVH group six patients died. The mortality was 40%.

**Table 1:**
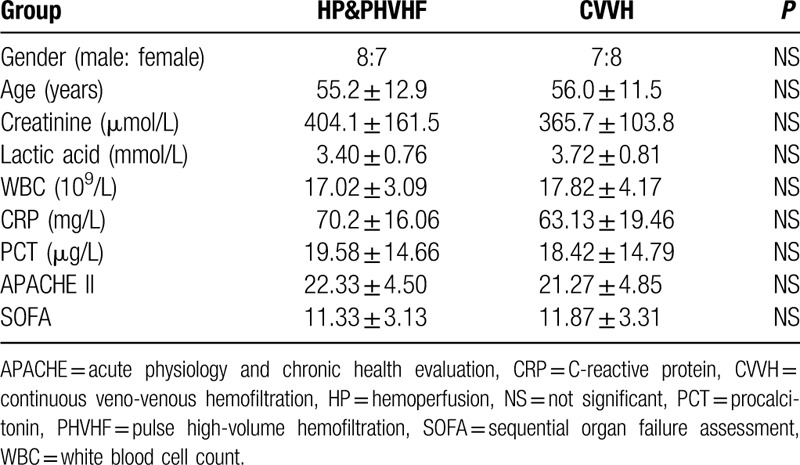
Patient demographics and baseline characteristics.

### 3.2 Changes in clinical manifestations, vital signs and laboratory test results

All the 30 patients received hemofiltration treatment at least for 72 h. In both groups, vital signs were significantly improved. Especially in combined group, the decrease of HR was more obvious than CVVH group (*P* < .05) (Table 2). Simultaneously, the laboratory test results such as white blood cells, CRP, Procalcitonin, lactic acid, serum creatinine in both of the two groups showed significant decrease (*P* < .05). Compared with CVVH group, the decrease of CRP PCT and WBC in HP&PHVHF group was more significant (*P* < .05). During the treatment, the acidosis and hypoxemia were effectively corrected, the internal environment was stable, and the electrolyte and PH values were within the normal range. APACHE II scores and SOFA scores were also decreased in both groups, and HP&PHVHF group was better than CVVH group (*P* < .05) (Table 3).

**Table 2:**
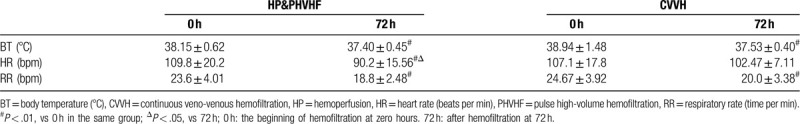
Changes of vital signs after treatment in two groups (mean ± SD, n = 15).

**Table 3:**
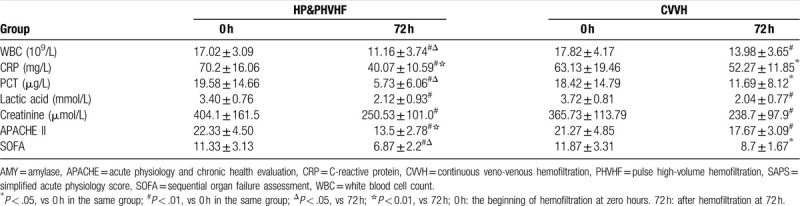
Laboratory and physiological variables before and after treatment in two groups (mean ± SD, n = 15).

### 3.3 Hemodynamic outcome

Norepinephrine was the first-choice vasopressor to maintain means arterial pressure above 85 mm Hg. No patients developed threatening hypotension during hemofiltration. In both groups, the doses of norepinephrine were decreased after 72 h of treatment. The combined group was decreased from 0.69 (0.15) to 0.25 (0.20) (*P* < .01), and the other group was decreased from 0.67 (0.17) to 0.39 (0.18) (*P* < .01). HP&PHVHF group was more obvious than CVVH group (*P* < .05).

### 3.4 Changes of plasma cytokine

**Figure 1:**
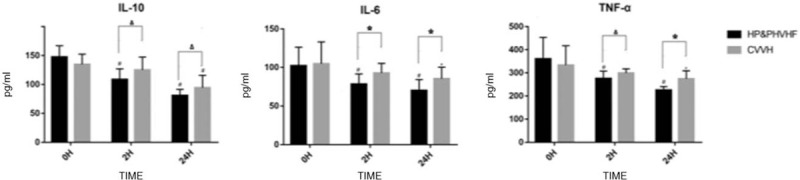
The levels of interleukin (IL)-6, IL-10 and tumor necrosis factor (TNF)-α before and after treatment in the two groups. Data are expressed as mean value ± standard deviation (SD) (pg/mL). ^∗^*P* < .05, vs pre-treatment; ^#^*P* < .01, vs pre-treatment; ^Δ^*P* < .05, vs end treatment; ^☆^*P* < .01, vs end-treatment.

Twenty-four hours after treatment, the levels of IL-10, IL-6, and TNF-α decreased in both groups (*P* < .05). The decrease of these inflammatory factors was more evident in the combined group (*P* < .05). Especially in combined group, after 2 h of hemoperfison, there was a significant reduction in these inflammatory factors (*P* < .01) (Fig. 1).

### 3.5 Side effects

Two patients with pulmonary infection have bloody phlegm during hemofiltration. Bleeding was controlled by reducing the administration of anticoagulant.

## 4 Discussion

Despite the great advances in the treatment of sepsis over the past few decades, severe sepsis and septic shock are still difficult to treat with poor prognosis and high mortality.^[9]^ In context of the pathogenesis of sepsis and septic shock, a comprehensive response and abnormal immune regulation to the infection have in recent years gained considerable interest.^[10]^ Sepsis is defined as the host's pathological response to infection. The immune system is activated rapidly after the onset of the severe infection, aiming to capture and eliminate the pathogen, and releases a large excess of pro- and anti-inflammatory cytokines, including IL-10, IL-6, TNF-α, and so on.^[11,12]^ This may lead to an uncontrolled, excessive inflammatory response acting like a cascade. In a few hours to several days the storm caused by inflammation will lead to vascular endothelial injury, extensive tissue damage, the distant organ complications and even death. There is vast evidence to show that the death was related to sepsis-induced immune disorders and the increased risk of secondary infections.^[13,14]^ Therefore, early nonspecific removal of excessive inflammatory mediators may control the progression of sepsis and improve prognosis.

CVVH, as a kind of continuous hemodialysis mode, by supplying the displacement fluid and ultrafiltration slowly, not only can clean out superfluous water and toxin smoothly, keep water, electrolyte and acid base balance, stable hemodynamics, but also can remove inflammatory mediators non-selectively, downregulate inflammatory response and restore the immunologic homeostasis.^[15]^ CVVH has been wildly used in critical diseases, including severe sepsis, septic shock, severe acute pancreatitis, adult respiratory distress syndrome, multiple organ dysfunction syndrome and so on.^[16]^ Previous studies have showed that CVVH is effective in septic shock.^[17,18]^ This study also provided evidence that CVVH is an effective treatment for septic shock, in accordance with previous studies.

High-volume hemofiltration is defined as application of ultrafiltration greater than what is used to support renal function. Large ultrafiltration flows were used to increase the elimination of medium-molecular-mass molecules which include many inflammatory mediators, and then can improve hemodynamics. Multiple animal and human studies have shown that HVHF is more beneficial on hemodynamics and survival.^[4,19–23]^ However, as a continuous modality, some obvious shortcomings of HVHF limit its clinical use. Continuous mega doses of hemofiltration solution require very intensive care, which is difficult to maintain over 24 h. Large dosage of replacement fluid increases the cost of treatment. Besides, after a few hours of hemofiltration, the removal efficiency of inflammatory factors can be greatly reduced with the decreasing of the saturation of membrane adsorption. In view of the above, Ronco advanced a new modality namely pulse HVHF (PHVHF) as an improved model of HVHF: A daily schedule of HVHF (85 mL/kg/h) for 6 to 8 h followed by CVVH (35 mL/kg/h) for the remaining time, which can lead to an average dose of approximately 48 mL/kg/h.^[5]^ In theory, the effect of pulse therapy can be maintained by the following standard CVVH and prevent post-treatment rebound from treatment interruption. In the study by Ronco, hemodynamics of septic shock patients was effectively improved by PHVHF. There was an apparent decline in the dose of noradrenaline during and at the end of the pulse therapy and the improvement of hemodynamics was maintained in the CVVH phase.^[22]^ In our previous study, we have applied PHVHF to the treatment of severe acute pancreatitis with MODS. The results confirmed that PHVHF can effectively relieve clinical symptoms, improve hemodynamics and improve prognosis.^[23]^

There are some other blood purification strategies for the treatment of septic shock. In terms of efficacy and security, the most promising of these is HP, which uses materials with high adsorptive properties.^[22]^ The molecules are attracted by the adsorptive surface through hydrophobic, ionic and van der Waals interaction when the blood circulates contact it. Inflammatory mediators may be removed from the blood by being bound to the adsorptive surface. Multiple human studies had demonstrated that HP can reduce inflammatory factors and improve survival of septic shock patients.^[24,25]^ But HP is not a renal replacement method, so the combination of HP and PHVHF contains multiple mechanisms of blood purification, including the strengthened adsorption and convection, and may have special advantages on the therapy of septic shock, but the clinical studies are scarce.

In this study, we used HP combined with PHVHF to treat septic shock. Our results showed that HR, CRP, PCT, WBC APACHE II score, and SOFA score declined dramatically in combined group compared with the CVVH group. We also found hemodynamic benefits for septic shock patients in this study. No patients developed threatening hypotension during hemofiltration. The doses of norepinephrine were also decreased after treatment, with more reduction in the combined group, which was in accordance with the result of the study by Ronco.^[22]^ The 28-day all-cause mortality in the combined group was 26.7%, and the CVVH group had a 40% mortality rate. CVVH has been proven to be an effective treatment for sepsis, and the combined treatment can further reduce mortality. May be despite the small sample size, there was no significant difference in mortality. This investigation established the fact that the combination of HP and PHVHF, fully utilizing different blood purification principles was well tolerated by septic shock patients and can improve hemodynamics and survival rate. This combination might be a safe and effectively modality and can be performed as a routine treatment model.

In this study, we also observed the changes of some inflammatory factors in the plasma, including TNF-α, IL-6, and IL-10. In both the two groups, IL-6, IL-10, and TNF-α all decreased in different degrees. In the combination therapy group, the decrease was more significant, especially after HP for 2 h. In previous studies, there were some controversy about the changes of plasma inflammatory factors levels during the continuous hemofiltration therapy.^[17,26–28]^ Some studies have found evidence for the removal of inflammatory mediators by continuous hemofiltration and reduce the plasma cytokine concentrations.^[28]^ In contrast, some studies suggested that there's no reduction, but cytokines were detected in ultrafiltrate.^[17,27]^ The severity of the disease, the duration of treatment intervention and the different ultrafiltration rates may affect the production, clearance and distribution of inflammatory factors. Circulating inflammatory mediators may just be the “tip of the iceberg,” and the decrease in plasma cannot accurately reflect the clearance rate by the blood purification.

Our study had a number of limitations. First, the number of cases involved in the study was limited. Second, the levels of inflammatory factors were only measured up to 24 h following treatment, and we did not measure them at later time points. Third, we did not detect the inflammatory factors in ultrafiltration to provide more evidence. In the further study, we will combine the changes of inflammatory factors in plasma and ultrafiltrate to scientifically expound the removal efficiency of blood purification.

## 5 Conclusions

In summary, HP combined with PHVHF appears to be a safe and effective technique, which contains multiple mechanisms of blood purification, including the strengthened adsorption and convection. It may have special advantages on the therapy for the patient suffering from septic shock and can be as an effective part of treatment by maintaining hemodynamics, balancing internal milieu and reducing the levels of inflammatory factors. The combination of two different principles of blood purification may be a tendency treatment of critical disease, including severe sepsis, severe acute pancreatitis, MODS and so on. Some of them are very interesting and have a chance of being included in clinical practice in the nearest future.

## Author contributions

**Conceptualization**: Laping Chu, Guangyao LI, Yafen Yu, Xiaoyan Bao, Hongyi Wei, Minhong Hu.

**Data curation**: Laping Chu, Guangyao LI, Xiaoyan Bao, Hongyi Wei, Minhong Hu

**Formal analysis**: Laping Chu, Yafen Yu.

**Funding acquisition**: Laping Chu

**Investigation**: Laping Chu, Guangyao LI,Xiaoyan Bao, Hongyi Wei

**Writing – original draft**: Laping Chu

**Writing – review & editing**: Laping Chu, Yafen Yu
